# Quantitative relaxometry using synthetic MRI could be better than T2-FLAIR mismatch sign for differentiation of IDH-mutant gliomas: a pilot study

**DOI:** 10.1038/s41598-022-13036-0

**Published:** 2022-06-02

**Authors:** Kazufumi Kikuchi, Osamu Togao, Koji Yamashita, Daichi Momosaka, Yoshitomo Kikuchi, Daisuke Kuga, Nobuhiro Hata, Masahiro Mizoguchi, Hidetaka Yamamoto, Toru Iwaki, Akio Hiwatashi, Kousei Ishigami

**Affiliations:** 1 Department of Clinical Radiology, Graduate School of Medical Sciences, Kyushu University, Fukuoka, Japan; 2 Department of Molecular Imaging and Diagnosis, Graduate School of Medical Sciences, Kyushu University, 3-1-1 Maidashi, Higashi-ku, Fukuoka, 812-8582 Japan; 3 National Hospital Organization, Kyushu Medical Center, Fukuoka, Japan; 4 Department of Neurosurgery, Graduate School of Medical Sciences, Kyushu University, Fukuoka, Japan; 5 Department of Anatomic Pathology, Graduate School of Medical Sciences, Kyushu University, Fukuoka, Japan; 6 Department of Neuropathology, Graduate School of Medical Sciences, Kyushu University, Fukuoka, Japan

**Keywords:** CNS cancer, CNS cancer

## Abstract

This study aimed to determine whether quantitative relaxometry using synthetic magnetic resonance imaging (SyMRI) could differentiate between two diffuse glioma groups with isocitrate dehydrogenase (IDH)-mutant tumors, achieving an increased sensitivity compared to the qualitative T2-fluid-attenuated inversion recovery (FLAIR) mismatch sign. Between May 2019 and May 2020, thirteen patients with IDH-mutant diffuse gliomas, including seven with astrocytomas and six with oligodendrogliomas, were evaluated. Five neuroradiologists independently evaluated the presence of the qualitative T2-FLAIR mismatch sign. Interrater agreement on the presence of the T2-FLAIR mismatch sign was calculated using the Fleiss kappa coefficient. SyMRI parameters (T1 and T2 relaxation times and proton density) were measured in the gliomas and compared by the Mann–Whitney U test. Receiver operating characteristic curve analysis was used to evaluate the diagnostic performance. The sensitivity, specificity, and kappa coefficient were 57.1%, 100%, and 0.60, respectively, for the qualitative T2-FLAIR mismatch sign. The two types of diffuse gliomas could be differentiated using a cutoff value of 178 ms for the T2 relaxation time parameter with 100% sensitivity, specificity, accuracy, and positive and negative predictive values, with an area under the curve (AUC) of 1.00. Quantitative relaxometry using SyMRI could differentiate astrocytomas from oligodendrogliomas, achieving an increased sensitivity and objectivity compared to the qualitative T2-FLAIR mismatch sign.

Isocitrate dehydrogenase (IDH) enzymes play a key role in the tumorigenesis of glioma. IDH mutations, including IDH1 and IDH2 mutations, were initially identified in glioblastoma^1^. A previous study revealed that IDH mutations were more frequently observed in diffuse low-grade gliomas, including both astrocytomas and oligodendrogliomas^2^. In low-grade gliomas, IDH mutation is an early event in tumor development that is associated with a malignant transformation involving a secondary glioblastoma^3^. IDH-mutant astrocytomas and IDH-mutant and 1p/19q-codeleted (the deletion of the short arm of chromosome 1 and the long arm of chromosome 19) oligodendrogliomas are classified as diffuse gliomas^4^. These two types of glioma have the same IDH mutation status; however, their prognoses vary^5^. IDH-mutant and 1p/19q-codeleted oligodendrogliomas are associated with better prognoses^6^ and respond better to chemotherapy or radiotherapy than IDH-mutant astrocytomas^7^. Thus, differential diagnosis is crucial for patient management.

In the differential diagnosis of these diffuse gliomas, the so-called “T2-fluid-attenuated inversion recovery (FLAIR) mismatch sign” was reported as a specific imaging marker for IDH-mutant astrocytomas^5^. This simple visual sign has a specificity of 100%; however, it has low sensitivity (12–51%)^5,8^ and is considered subjective since its diagnosis is based on its appearance in imaging evaluations.

We hypothesized that quantitative relaxometry would show increased sensitivity and objectivity compared to the qualitative T2-FLAIR mismatch sign. Synthetic MRI (SyMRI) can directly evaluate T1 and T2 relaxation times and proton density (PD) values during the clinical scan time and hence was considered for quantitative relaxometry^9^. To the best of our knowledge, no study has quantitatively evaluated the diagnostic accuracy of relaxometry in patients with IDH-mutant lower-grade gliomas.

Hence, the purpose of this study was to determine whether quantitative relaxometry using SyMRI could differentiate these diffuse gliomas, achieving an increased sensitivity when compared to the qualitative T2-FLAIR mismatch sign.

## Results

### Patient demographics and characteristics

**Figure 1:**
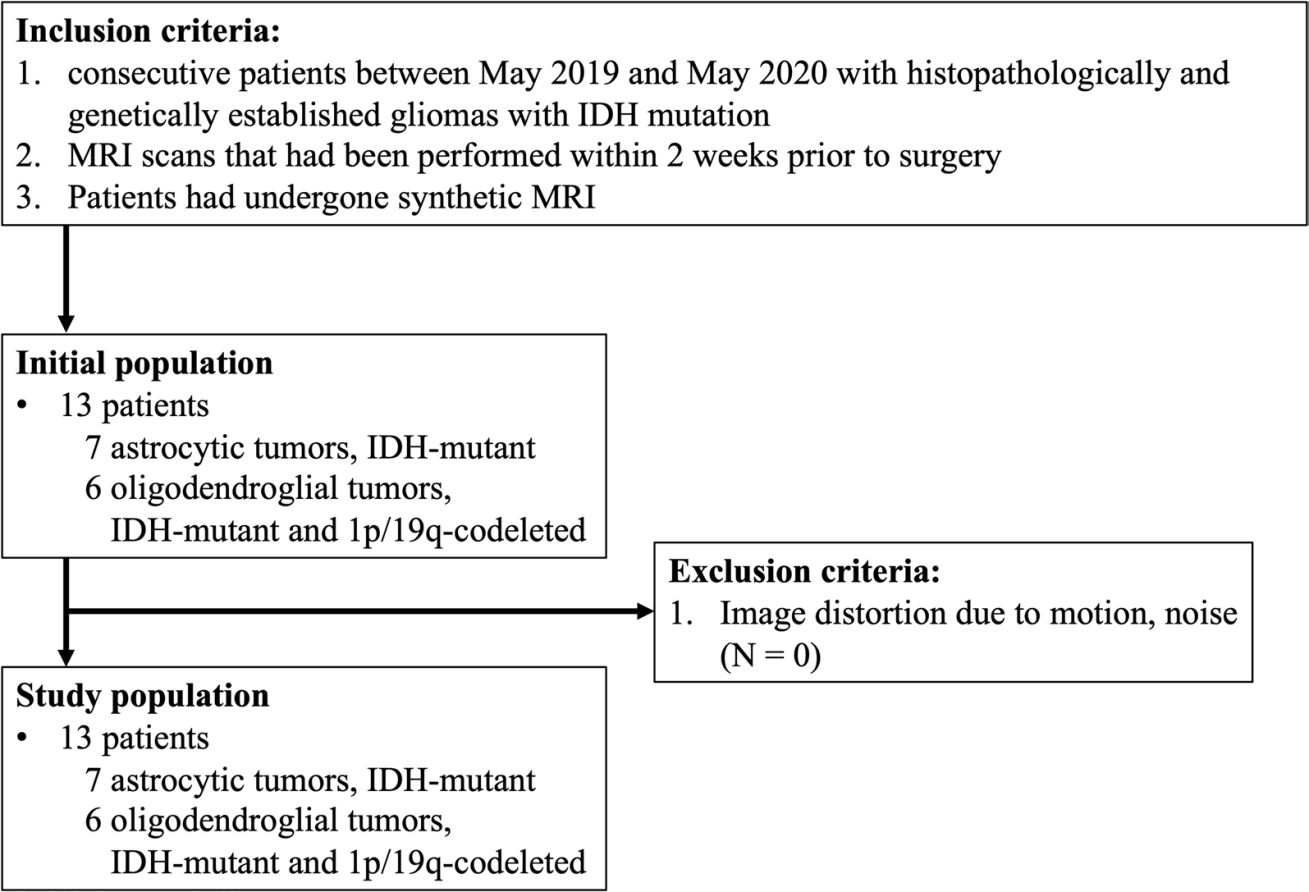
Patient selection flowchart.

A total of 13 patients (median age, 43 years; interquartile range, 36.8–51.2 years; six male and seven female patients) were included in this study. There were seven patients with IDH-mutant astrocytomas (range, median age: 29–49, 41 years; three males and four females), and six with IDH-mutant and 1p/19q-codeleted oligodendrogliomas (37–63, median age: 48 years; three males and three females). An overview of the patient demographics and characteristics is presented in Table 1, and the patient selection process is depicted in Fig. 1. There was a significant difference observed in the T2-FLAIR mismatch sign between the two tumor groups (*p* = 0.02). The patients’ demographics and pathological diagnoses, based on the 2016 World Health Organization (WHO) classification^4^, were as follows: seven patients with astrocytomas, including four with diffuse astrocytomas (WHO grade II; age, 33–44 years; two males and two females), one with anaplastic astrocytoma (WHO grade III; age, 29 years; one male), and two with glioblastomas (WHO grade IV; age, 45 and 49 years; two females), and six patients with oligodendrogliomas, including four with oligodendrogliomas (WHO grade II; age, 40–63 years; two males and two females) and two with anaplastic oligodendrogliomas (WHO grade III; age, 37 and 43 years; one male and one female). All the patients were genetically verified as having IDH-mutant type tumors. Patients with postoperative recurrences (three astrocytomas and two oligodendrogliomas) were included, and the recurrences were confirmed in the pathological specimens obtained during the second surgery.

**Table 1:** Patient demographics and characteristics.

Parameter	Astrocytic tumor, IDH-mutant	Oligodendroglial tumor, IDH-mutant and 1p/19q-codeleted	All gliomas	^a^*p* Value
No. of patients	7	6	13	…
Median age (years)	41 (29–49) [33.8, 45.8]	48 (37–63) [39.8, 58.4]	43 (29–63) [36.8, 51.2]	0.07
Sex (M/W)	3/4	3/3	6/7	0.79
WHO grade (II/III/IV)	4/1/2	4/2/0	8/3/2	0.32
Tumor size (mm^2^)	808 (356–2379) [493, 1746]	1477 (366–4640) [911, 2482]	1380 (356–4640) [510, 1755]	0.27
Location (F/P/I)	4/1/2	5/0/1	9/1/3	0.50
Enhancement (+/−)	1/6	2/4	3/10	0.42
Calcification (+/−)	0/7	1/5	1/12	0.26
Cystic component (+/−)	2/5	2/4	4/9	0.85
Hemorrhage (+/−)	2/5	2/4	4/9	0.85
T2-FLAIR mismatch sign (+/−)	4/3	0/6	4/9	0.03

Data in parentheses are the range, and data in brackets are the interquartile range.

*1p/19q-codeleted* = deletion of the short arm of chromosome 1 and long arm of chromosome 19, *F* = frontal lobe, *I* = insula, *FLAIR* = fluid-attenuated inversion recovery, *IDH* = isocitrate dehydrogenase, *M* = men, *P* = parietal lobe, *W* = women, *WHO* = World Health Organization.

^a^Chi-square test.

### Qualitative evaluation

Table 2 shows the qualitative results from the five neuroradiologists. The mean sensitivity, specificity, accuracy, positive predictive value (PPV), and negative predictive value (NPV) were 57.1%, 100%, 76.9%, 100%, and 67.0%, respectively. Moderate interrater agreement was observed (kappa value = 0.60).

**Table 2:** Qualitative evaluation based on the T2-FLAIR mismatch sign among the 5 radiologists.

	Reader 1	Reader 2	Reader 3	Reader 4	Reader 5	Average
Sensitivity (%)	57.1 (4/7) [18.4, 90.1]	42.9 (3/7) [9.9, 81.6]	57.1 (4/7) [18.4, 90.1]	57.1 (4/7) [18.4, 90.1]	71.4 (5/7) [29.0, 96.3]	57.1
Specificity (%)	100.0 (6/6) [54.1, 100]	100.0 (6/6) [54.1, 100]	100.0 (6/6) [54.1, 100]	100.0 (6/6) [54.1, 100]	100.0 (6/6) [54.1, 100]	100.0
Accuracy (%)	76.9 [46.2, 95.0]	69.2 [38.6, 90.9]	76.9 [46.2, 95.0]	76.9 [46.2, 95.0]	84.6 [54.6, 98.1]	76.9
PPV 9%)	100.0 [not applicable]	100.0 [not applicable]	100.0 [not applicable]	100.0 [not applicable]	100.0 [not applicable]	100.0
NPV (%)	66.7 [46.0, 82.5]	60.0 [44.1, 74.0]	66.7 [46.0, 82.5]	66.7 [46.0, 82.5]	75.0 [48.2, 90.6]	67.0

*NPV* negative predictive value, *PPV* positive predictive value.

Data in parentheses are numerators/denominators; data in brackets are 95% confidence intervals.

### Quantitative evaluation

**Figure 2:**
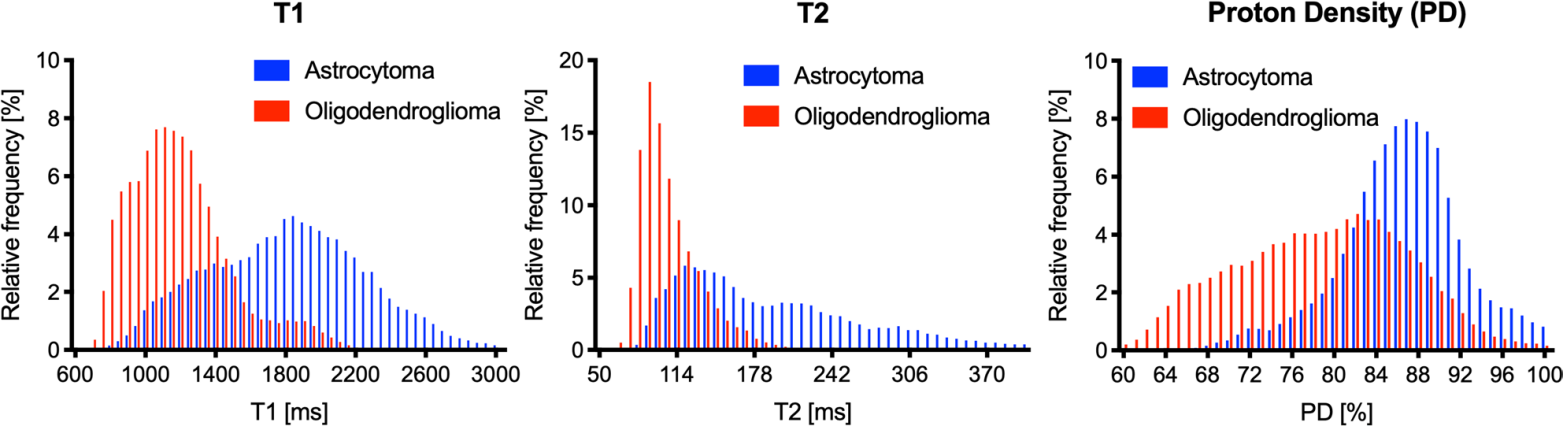
Histograms of T1 and T2 relaxation times and proton density (PD) between IDH-mutant astrocytomas and IDH-mutant and 1p/19q-codeleted oligodendrogliomas. All parameters (T1 and T2 relaxation times and PD) in astrocytomas exhibit a slight rightward shift (‘+’) relative to those in oligodendrogliomas.

Figure 2 and Supplementary Table S1 show the histograms of each parameter over all the pixels in the tumor regions of interest (ROIs). T1 and T2 relaxation times and PDs from the astrocytomas all exhibited a slight rightward shift relative to those from the oligodendrogliomas. T1 and T2 relaxation times and PDs were larger for astrocytomas than for oligodendrogliomas (mean ± standard deviation, *p* value: 2047 ± 454 vs. 1290 ± 274 ms, *p* < 0.0001 for T1 relaxation time; 254 ± 124 vs. 109 ± 16 ms, *p* < 0.0001 for T2 relaxation time; 89.4 ± 5.3 vs. 80.0 ± 4.4%, *p* = 0.01 for PD, respectively). There were also significant differences in the 10–90th percentiles for T1 and T2 relaxation times and PDs (all *p* < 0.05). Table 3 shows the diagnostic performance in differentiating the two glioma groups; the most useful values of each parameter are shown in Table 3. The entire set of our results is shown in Supplementary Table S2. The two types of diffuse gliomas could be differentiated using a cutoff value of 178 ms for the T2 relaxation time parameter with 100% sensitivity, 100% specificity, 100% accuracy, and 100% PPV and NPV, with an area under the curve (AUC) of 1.00.

**Table 3:** Diagnostic performance of parameters in differentiating between IDH-mutant astrocytic tumors and IDH-mutant and 1p/19q-codeleted oligodendroglial tumors.

Parameters	Sensitivity (%)	Specificity (%)	Accuracy (%)	PPV (%)	NPV (%)	Cutoff	AUC
*T1 (ms)*							
50th percentile	100.0 (7/7) [59.0, 100.0]	83.3 (5/6) [35.9, 99.6]	92.3 [64.0, 99.8]	87.5 [53.9, 97.7]	100.0 [not applicable]	1332	0.95
90th percentile	85.7 (6/7) [42.1, 99.6]	100.0 (6/6) [54.1, 100.0]	92.3 [64.0, 99.8]	100.0 [not applicable]	85.7 [49.4, 97.4]	2290	0.95
Mean	100.0 (7/7) [59.0, 100.0]	83.3 (5/6) [35.9, 99.6]	92.3 [64.0, 99.8]	87.5 [53.9, 97.7]	100.0 [not applicable]	1407	0.95
*T2 (ms)*							
10th percentile	100.0 (7/7) [59.0, 100.0]	100.0 (6/6) [54.1, 100.0]	100.0 [75.3, 100.0]	100.0 [not applicable]	100.0 [not applicable]	100	1.00
50th percentile	100.0 (7/7) [59.0, 100.0]	100.0 (6/6) [54.1, 100.0]	100.0 [75.3, 100.0]	100.0 [not applicable]	100.0 [not applicable]	148	1.00
Mean	100.0 (7/7) [59.0, 100.0]	100.0 (6/6) [54.1, 100.0]	100.0 [75.3, 100.0]	100.0 [not applicable]	100.0 [not applicable]	178	1.00
*PD (%)*							
10th percentile	71.4 (5/7) [29.0, 96.3]	100.0 (6/6) [54.1, 100.0]	84.6 [54.6, 98.1]	100.0 [not applicable]	75.0 [48.2, 90.6]	81.8	0.90
25th percentile	71.4 (5/7) [29.0, 96.3]	100.0 (6/6) [54.1, 100.0]	84.6 [54.6, 98.1]	100.0 [not applicable]	75.0 [48.2, 90.6]	84.0	0.90
50th percentile	71.4 (5/7) [29.0, 96.3]	100.0 (6/6) [54.1, 100.0]	84.6 [54.6, 98.1]	100.0 [not applicable]	75.0 [48.2, 90.6]	86.4	0.90
Mean	71.4 (5/7) [29.0, 96.3]	100.0 (6/6) [54.1, 100.0]	84.6 [54.6, 98.1]	100.0 [not applicable]	75.0 [48.2, 90.6]	86.7	0.90

*AUC* area under the curve, *IDH* isocitrate dehydrogenase, *NPV* negative predictive value, *PD* proton density, *PPV* positive predictive value.

Data in parentheses are numerators/denominators; data in brackets are 95% confidence intervals.

**Figure 3:**
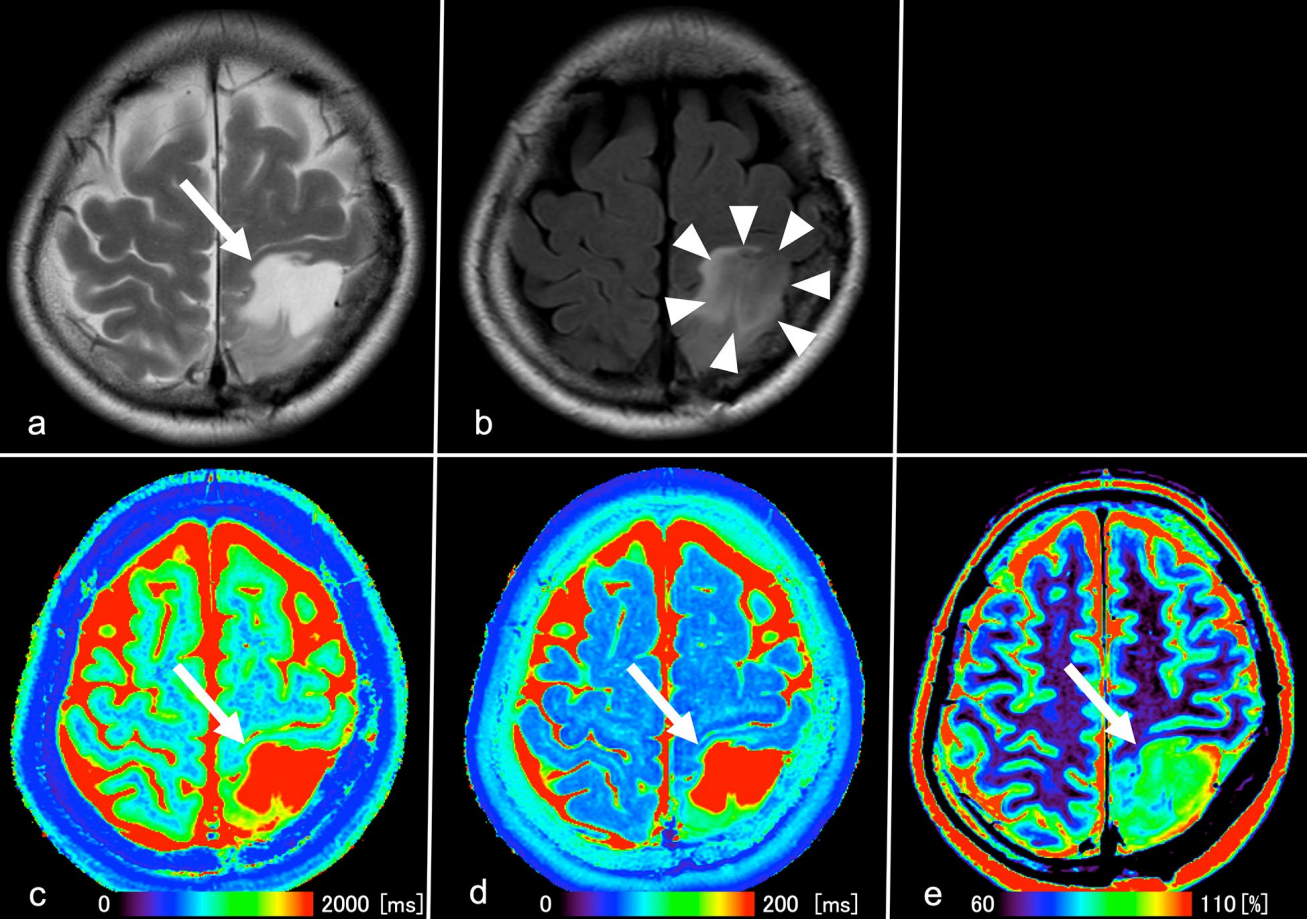
Images from a 49-year-old woman with IDH-mutant diffuse astrocytoma (WHO grade II). (**a**) T2WI shows a heterogeneous T2-prolonged mass in the left parietal lobe (arrow). (**b**) FLAIR shows partial signal suppression, indicating a T2-FLAIR mismatch sign (arrowheads). (**c**), (**d**), (**e**), T1 and T2 relaxation time and proton density (PD) maps derived from SyMRI show T1 (2436 ms*) and T2 (287 ms*) relaxation time prolongations and increased PD (94.9%*) (arrows) in the tumor. *Each value is expressed as the mean.

**Figure 4:**
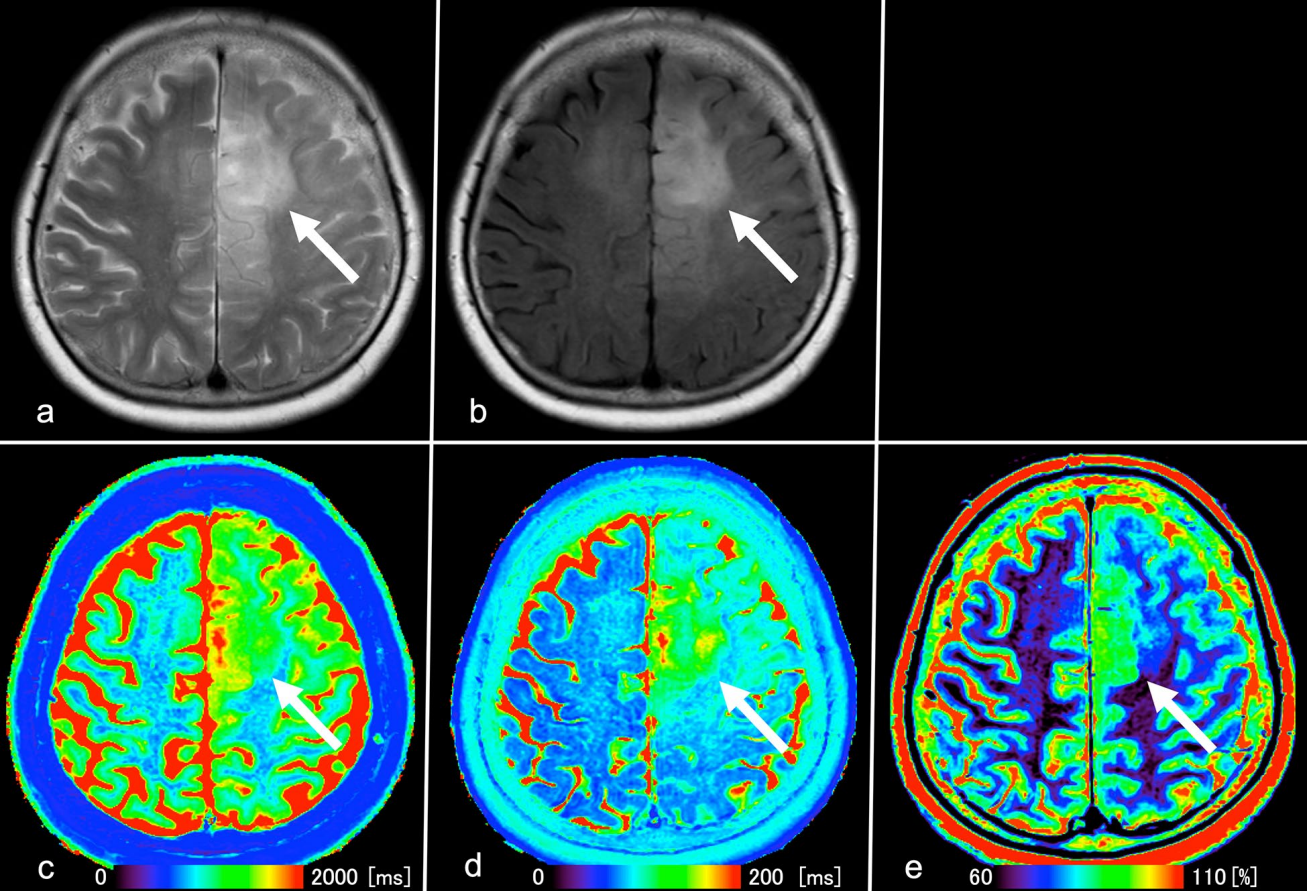
Images from a 63-year-old woman with IDH-mutant and 1p/19q-codeleted oligodendroglioma (WHO grade II). (**a**) T2WI shows a heterogeneous, poorly circumscribed mass in the left frontoparietal lobes. (**b**) FLAIR shows no signal suppression in the tumor, indicating no T2-FLAIR mismatch sign (arrow). (**c**), (**d**), (**e**), T1 and T2 relaxation time and proton density (PD) maps derived from SyMRI show mild T1 (1119 ms*) and T2 (103 ms*) relaxation time prolongations and mildly increased PD (76.8%*) (arrows) in the tumor. *Each value is expressed as the mean.

Figures 3 and 4 show representative images of patients with astrocytoma and oligodendroglioma, respectively.

## Discussion

As anticipated, our results confirmed that quantitative relaxometry using SyMRI could differentiate IDH-mutant gliomas, leading to an increased sensitivity compared to the qualitative T2-FLAIR mismatch sign. The qualitative results showed high specificity; however, the sensitivity was low, and the interrater agreement was moderate. Quantitative results showed that there were significant differences in the 10–90th percentiles and the mean between the gliomas for all SyMRI parameters. The receiver operating characteristic (ROC) curve analysis revealed that the T2 relaxation time 10th and 50th percentile and mean values showed the highest diagnostic ability in differentiating gliomas.

Previous studies have reported that the T2-FLAIR mismatch sign has been shown to predict IDH-mutant astrocytoma with 95–100% specificity^10,11^; however, the sensitivity has been low at 22–51%^8,10,12^. The interrater agreement has been shown to have a wide range, κ = 0.38–0.88^5,8,10,11^. Jain et al.^13^ mentioned that strict application of the criteria is necessary to maintain high specificity, which tends to result in low sensitivity. Our results are consistent with those of these previous results. The low interrater agreement is probably due to the binary scoring system used, whereas subtle changes may result in different interpretations across readers^8^.

Our quantitative results showed long T1 and T2 relaxation times and increased PDs within astrocytomas compared to oligodendrogliomas. To our knowledge, this is the first study to quantitatively evaluate IDH-mutant gliomas using SyMRI. These prolonged T1 and T2 relaxation times imply fluid changes in the IDH-mutant astrocytomas. Deguchi et al. revealed that abundant microcysts were observed upon hematoxylin–eosin staining of specimens from the T2-FLAIR mismatched region in IDH-mutant astrocytomas^11^, which may reflect the T1 and T2 relaxation time prolongations. Kinoshita et al. evaluated gliomas quantitatively using MP2RAGE images calculated via Bayesian inference modeling and showed that the T2-FLAIR mismatch region exhibited extremely long T1 and T2 relaxation times^12^. Their results also support fluid changes within IDH-mutant astrocytomas. Our quantitative results showed that there were significant differences in the mean values for all SyMRI parameters between the two glioma groups. These findings may support the clinical utility of SyMRI to diagnose gliomas because only the placement of a simple ROI in the tumor is required, not a histogram analysis. The 10th and 50th percentile values are not as good as the mean value owing to the simplicity of the measurement for actual clinical use and the fact that they are supplementary indicators. Furthermore, as expected, quantitative evaluation significantly increased the sensitivity compared to the qualitative T2-FLAIR mismatch sign. Advanced MR techniques, such as dynamic perfusion MRI^14^ and MR spectroscopy^15^, have also revealed differences between IDH-mutant and wild-type gliomas. However, dynamic perfusion MRI requires contrast media, and 2-hydroxyglutarate MR spectroscopy has been shown to be associated with false-positive cases with intratumoral hemorrhage^16^. In addition, 2-hydroxyglutarate detection with MR spectroscopy is technically challenging due to the spectral overlap of 2-hydroxyglutarate with background metabolites^17^.

This study has several limitations. First, the sample size was small, and our study included postoperative cases. Although postoperative changes may affect the results of the qualitative evaluation, the quantitative evaluation was still useful to differentiate the gliomas in this study. Our results demonstrate that different subtypes of diffuse gliomas have different relaxation properties. Second, our study did not evaluate IDH-wild-type astrocytomas. It would be desirable to proceed with a subsequent study including patients with IDH-wild-type astrocytomas. Third, we did not include the whole tumor volume in the histogram analysis. Instead, we used the maximum section of the tumor, with its boundary defined by the hyperintensity on T2-weighted imaging (T2WI). However, in previous studies on the T2-FLAIR mismatch sign, whole-volume histogram analysis was not performed, and only the maximum section of the tumor was utilized. Simple evaluation based on the maximum-sized slice of the tumor could be sufficient since the T2-FLAIR mismatch sign criteria are designed such that high specificity is maintained rather than increasing sensitivity by employing these strict criteria^13^. If quantitative volumetry were conducted, a histogram of the entire tumor would be provided, and the optimal cutoff value would presumably be different from the present results; however, it is likely that astrocytomas would have shown longer T1 and T2 values than oligodendrogliomas.

In conclusion, relaxometry using SyMRI could differentiate IDH-mutant astrocytomas from IDH-mutant and 1p/19q-codeleted oligodendrogliomas. Quantitative relaxometry could increase sensitivity and objectivity compared to the qualitative T2-FLAIR mismatch sign; therefore, this objective evaluation provides a helpful, noninvasive diagnostic method for differentiating IDH-mutant gliomas. Prospective multicenter validation is needed to confirm our findings.

## Materials and methods

### Patient selection and clinical data

All patients consecutively diagnosed with glioma at our institution from May 2019 to May 2020 were eligible for this study. Inclusion criteria were (1) patients with IDH-mutant and/or 1p/19q-codeleted glioma based on the WHO classification^4^; (2) MRI scans that had been performed within two weeks before surgery; and (3) patients for whom SyMRI was acquired. The exclusion criterion was image distortion, such as motion artifacts or noise.

### Ethics approval

The institutional review board of Kyushu University Hospital approved this retrospective study, and the requirement for informed consent was waived. All methods were performed in accordance with the relevant guidelines and regulations.

### Histopathological analysis

All tissue samples were analyzed based on the WHO 2016 classification^4^. Immunohistochemistry for IDH1 R132H, ATRX, p53, and Ki67 was routinely performed. IDH1/2 was analyzed using high-resolution melting with DNA extracted from frozen tissue samples. The 1p/19q codeletions were evaluated using a microsatellite-based loss of heterozygosity analysis with 18 markers to detect the loss of the entire chromosome arm^19^.

### MRI

MRI was performed using a 3 T MR scanner (Ingenia 3.0 T CX; Philips Healthcare, Best, Netherlands) with a 15-channel head coil. Quantitative MRI was performed using the two-dimensional axial quantification of relaxation times and PDs by the multiecho acquisition of a saturation recovery using a turbo spin-echo readout (QRAPMASTER) pulse sequence with two echo times (TEs; 13 and 100 ms) and four delay times to generate eight real images and eight imaginary images^9^. The other parameters included repetition time (TR), 4831 ms; flip angle (FA), 90°; number of excitations (NEX), 1; sensitivity encoding factor, 2.2; field of view (FOV), 230 × 189 (recon. 230^2^) mm^2^; matrix, 512^2^; echo-train length, 10; thickness/gap, 4.0/1.0 mm; 30 slices, and scan time 6 min 36 s. Quantification map acquisition was performed with SyMRI software (Version 19.0; SyMRI, Linköping, Sweden). Standard MR sequences (T1-weighted imaging [T1WI], T2WI, FLAIR, and contrast-enhanced T1WI) were also obtained. The sequence parameters of the two-dimensional axial T2WI and FLAIR sequences were as follows: T2WI—TR/TE, 3000/80 ms; FA, 90°; NEX, 1; FOV, 230^2^ mm^2^; matrix, 512 × 375 (recon. 512^2^); echo-training length, 15; thickness/gap, 5.0/1.0 mm; 22 slices, and scan time, 2 min 36 s, and FLAIR—TR/TE/TI, 10,000/120/2700 ms; FA, 180°; NEX, 1; FOV, 230 × 207 (recon. 230^2^) mm^2^; matrix, 320 × 228 (recon. 512^2^); echo-training length, 27; thickness/gap, 5.0/1.0 mm; 22 slices, and scan time, 3 min.

### Qualitative evaluation

Five board-certified neuroradiologists (with 23, 21, 19, 8, and 6 years of experience) were blinded to the patient information of the evaluated T2-FLAIR mismatch sign^5,8^. The T2-FLAIR mismatch sign was defined by the presence of 2 distinct MRI features as follows^5^:

1. The tumor displayed a complete or nearly complete and nearly homogeneous hyperintense signal on T2WI.
2. The tumor displayed a relatively hypointense signal on the FLAIR sequence except for a hyperintense peripheral rim.

These two criteria should be strictly used to maintain high specificity for the diagnosis of IDH-mutant astrocytomas^13^. Jain et al.^13^ introduced additional imaging features aiding in the accurate identification of the T2-FLAIR mismatch sign:

(3) Necrotic cavities do not represent the T2-FLAIR mismatch sign; small cysts do not meet the criteria for the T2-FLAIR mismatch sign.
(4) The T2-FLAIR mismatch sign is typically accompanied by little or no contrast enhancement.
(5) The degree of FLAIR signal suppression could be inhomogeneous within the tumor.
(6) Common imaging correlates include homogeneous hypointensity on noncontrast T1WI, markedly elevated apparent diffusion coefficient values, low blood volume on perfusion maps, and diffuse hypodensity on CT.

After independent data collection, the interreader agreement was calculated, and discordant results for the T2-FLAIR mismatch sign were resolved by consensus^5^. Five radiologists read both the T2WI and FLAIR images based on whether the T2-FLAIR mismatch sign was present or absent. The sensitivity, specificity, PPV, NPV, and accuracy were calculated.

### Quantitative evaluation

The DICOM data of the T1 and T2 relaxation time and PD maps were extracted by SyMRI software. We used a single maximum section of each tumor for the ROI analysis on the T2-prolonged region in the tumor using an ImageJ plugin (ImageJ/Fiji; version 2.0.0-rc-59/1.51 k, National Institutes of Health, Bethesda, MD). The maximum section of the tumor was visually decided as the largest orthogonal cross product of the tumor on the axial T2/FLAIR scans^20^. Using the ROI manager tool of ImageJ/Fiji, the ROI mask from the T2-prolonged region on conventional T2WI scans was copied and placed on each parameter map (T1 and T2 relaxation times and PD) to obtain pixel-by-pixel values for the histogram analyses. The 10th, 25th, 50th, 75th, and 90th percentiles and the mean, skewness, and kurtosis of each parameter were recorded from the histograms.

### Statistical analysis

Chi-square tests were used to compare the patients' categorical variables (e.g., sex, WHO grade, tumor location, presence or absence of enhancement, calcification, cyst, hemorrhage, T2-FLAIR mismatch sign). In the qualitative evaluation, the interrater agreement for the T2-FLAIR mismatch sign among the five observers was evaluated using Fleiss's kappa coefficient^21^. The kappa value was interpreted as follows: almost perfect agreement, 1.00–0.81; substantial agreement, 0.80–0.61; moderate agreement, 0.60–0.41; fair agreement, 0.40–0.21; slight agreement, 0.20–0.01; and poor agreement, < 0^22^. In the quantitative evaluation, the percentiles, mean, skewness, and kurtosis of each parameter (i.e., T1 and T2 relaxation times and PD) were compared between astrocytomas and oligodendrogliomas by the Mann–Whitney *U* test. The diagnostic performance of each parameter was evaluated by ROC curve analysis. The comparison of simulated T2-FLAIR mismatch signs was made by the Mann–Whitney *U* test. All statistical analyses were performed using commercial software programs (JMP, version 15.0.0; SAS Institute, Cary, NC, USA; Prism 7.0, GraphPad Software, La Jolla, CA, USA). *P* values < 0.05 were considered statistically significant.

## Supplementary Information

Supplementary Information.

## Data Availability

The datasets generated during and/or analyzed during the current study are available from the corresponding author on reasonable request.
