# Supplementary material for: Quantitative relaxometry using synthetic MRI could be better than T2-FLAIR mismatch sign for differentiation of IDH-mutant gliomas: a pilot study
Source: Sci Rep. 2022 Jun 2;12:9197. doi: 10.1038/s41598-022-13036-0 (PMC9163057; doi:10.1038/s41598-022-13036-0)
Supplement: Supplementary file 1 — Supplementary Information. [file 41598_2022_13036_MOESM1_ESM.docx]

**Quantitative Relaxometry Using Synthetic MRI Could Be Better Than T2-FLAIR Mismatch Sign for Differentiation of IDH-Mutant Gliomas: A Pilot Study**

Kazufumi Kikuchi^1^, M.D., Ph.D., Osamu Togao^2*^, M.D., Ph.D., Koji Yamashita^3^, M.D., Ph.D., Daichi Momosaka^1^, M.D., Ph.D., Yoshitomo Kikuchi^1^, M.D., Daisuke Kuga^4^, M.D., Ph.D., Nobuhiro Hata^4^, M.D., Ph.D., Masahiro Mizoguchi^4^, M.D., Ph.D., Hidetaka Yamamoto^5^, M.D., Ph.D., Toru Iwaki^6^, M.D., Ph.D., Akio Hiwatashi^1^, M.D., Ph.D., Kousei Ishigami^1^, M.D., Ph.D.

^1^Department of Clinical Radiology, Graduate School of Medical Sciences, Kyushu University, Fukuoka, Japan

^2^Department of Molecular Imaging and Diagnosis, Graduate School of Medical Sciences, Kyushu University, Fukuoka, Japan

^3^National Hospital Organization, Kyushu Medical Center, Fukuoka, Japan

^4^Department of Neurosurgery, Graduate School of Medical Sciences, Kyushu University, Fukuoka, Japan

^5^Department of Anatomic Pathology, Graduate School of Medical Sciences, Kyushu University, Fukuoka, Japan

^6^Department of Neuropathology, Graduate School of Medical Sciences, Kyushu University, Fukuoka, Japan

*Corresponding Author:

Osamu Togao, M.D., Ph.D.

Department of Molecular Imaging and Diagnosis

Graduate School of Medical Sciences

Kyushu University

3-1-1 Maidashi, Higashi-ku, Fukuoka 812-8582, Japan

Tel: 81-92-642-5695

Fax: 81-92-642-5708

E-mail: togao.osamu.595@m.kyushu-u.ac.jp

**Data availability.** The datasets generated during and/or analyzed during the current study are available from the corresponding author on reasonable request.

**Supplementary Table S1.** Comparison of the parameters between astrocytic tumor, IDH-mutant vs. oligodendroglioma, IDH-mutant and 1p/19q-codeleted

| **Parameter** | **Astrocytic tumor**  **IDH-mutant** | **Oligodendroglial tumor**  **IDH-mutant and 1p/19q-codeleted** | **^a^*P*-value** |
| --- | --- | --- | --- |
| T1 [ms] |  |  |  |
| 10^th^ percentile | 1517 (1170–1864) | 966 (733–1199) | 0.01 |
| 25^th^ percentile | 1685 (1327–2042) | 1106 (819–1392) | 0.01 |
| 50^th^ percentile | 1921 (1557–2286) | 1262 (959–1564) | < 0.0001 |
| 75^th^ percentile | 2458 (1646–3269) | 1458 (1148–1768) | < 0.0001 |
| 90^th^ percentile | 2677 (1947–3408) | 1629 (1284–1974) | < 0.0001 |
| Mean | 2047 (1628–2467) | 1290 (1002–1578) | < 0.0001 |
| Skewness | 0.57 (0.16–0.98) | 1.03 (0.36–1.69) | 0.29 |
| Kurtosis | 0.49 (–0.75–1.72) | 3.94 (–0.57–8.46) | 0.10 |
| T2 [ms] |  |  |  |
| 10^th^ percentile | 138 (96–179) | 84 (76–92) | < 0.0001 |
| 25^th^ percentile | 160 (114–207) | 94 (81–106) | < 0.0001 |
| 50^th^ percentile | 192 (141–243) | 105 (89–121) | < 0.0001 |
| 75^th^ percentile | 339 (88–589) | 119 (97–141) | < 0.0001 |
| 90^th^ percentile | 460 (35–885) | 136 (107–165) | 0.01 |
| Mean | 254 (139–369) | 109 (92–126) | < 0.0001 |
| Skewness | 1.41 (-0.48 to 3.31) | 2.76 -0.63 to 6.14) | 0.23 |
| Kurtosis | 6.75 -0.836 to 21.86) | 24.68 (-19.81 to 69.16) | 0.29 |
| PD [%] |  |  |  |
| 10^th^ percentile | 82.1 (77.2–87.1) | 71.7 (65.6–77.8) | 0.01 |
| 25^th^ percentile | 85.5 (81.1–89.8) | 75.4 (69.6–81.2) | 0.01 |
| 50^th^ percentile | 89.6 (84.8–94.4) | 79.9 (75.1–84.7) | 0.01 |
| 75^th^ percentile | 93.3 (87.8–98.8) | 84.8 (80.9–88.8) | 0.02 |
| 90^th^ percentile | 97.1 (90.2–104.0) | 88.5 (84.2–92.8) | 0.02 |
| Mean | 89.4 (84.5–94.3) | 79.9 (75.3–84.6) | 0.01 |
| Skewness | -0.4323 (–1.25–0.38) | -0.44 (-1.23 to 0.35) | 0.73 |
| Kurtosis | 3.01 (-2.03 to 8.05) | 1.99 (-2.70 to 6.69) | 0.36 |

Data are expressed as mean values and 95% confidence intervals.

*PD*, proton density

^a^Mann–Whitney U-test

**Supplementary Table 2.** Diagnostic performance of the parameters differentiating between astrocytic tumor, IDH-mutant and oligodendroglioma, IDH-mutant and 1p/19q-codeleted

| **Parameter** | **Sensitivity [%]** | **Specificity [%]** | **Accuracy [%]** | **PPV [%]** | **NPV [%]** | **Cutoff** | **AUC** |
| --- | --- | --- | --- | --- | --- | --- | --- |
| T1 [ms] |  |  |  |  |  |  |  |
| 10^th^ percentile | 71.4 | 100.0 | 84.6 | 100.0 | 75.0 | 1373 | 0.93 |
| 25^th^ percentile | 100.0 | 83.3 | 92.3 | 87.5 | 100.0 | 1204 | 0.93 |
| 50^th^ percentile | 100.0 | 83.3 | 92.3 | 87.5 | 100.0 | 1332 | 0.95 |
| 75^th^ percentile | 71.4 | 100.0 | 84.6 | 100.0 | 75.0 | 2107 | 0.93 |
| 90^th^ percentile | 85.7 | 100.0 | 92.3 | 100.0 | 85.7 | 2290 | 0.95 |
| Mean | 100.0 | 83.3 | 92.3 | 87.5 | 100.0 | 1407 | 0.95 |
| Skewness | 42.9 | 100.0 | 69.2 | 100.0 | 60.0 | 0.38 | 0.69 |
| Kurtosis | 100.0 | 66.7 | 84.6 | 77.8 | 100.0 | 2.21 | 0.79 |
| T2 [ms] |  |  |  |  |  |  |  |
| 10^th^ percentile | 100.0 | 100.0 | 100.0 | 100.0 | 100.0 | 100 | 1.00 |
| 25^th^ percentile | 85.7 | 100.0 | 92.3 | 100.0 | 85.7 | 116 | 0.98 |
| 50^th^ percentile | 100.0 | 100.0 | 100.0 | 100.0 | 100.0 | 148 | 1.00 |
| 75^th^ percentile | 85.7 | 100.0 | 92.3 | 100.0 | 85.7 | 210 | 0.98 |
| 90^th^ percentile | 85.7 | 100.0 | 92.3 | 100.0 | 85.7 | 235 | 0.93 |
| Mean | 100.0 | 100.0 | 100.0 | 100.0 | 100.0 | 178 | 1.00 |
| Skewness | 71.4 | 83.3 | 76.9 | 83.3 | 71.4 | 0.80 | 0.71 |
| Kurtosis | 71.4 | 83.3 | 76.9 | 83.3 | 71.4 | 0.37 | 0.69 |
| PD [%] |  |  |  |  |  |  |  |
| 10^th^ percentile | 71.4 | 100.0 | 84.6 | 100.0 | 75.0 | 81.8 | 0.90 |
| 25^th^ percentile | 71.4 | 100.0 | 84.6 | 100.0 | 75.0 | 84.0 | 0.90 |
| 50^th^ percentile | 71.4 | 100.0 | 84.6 | 100.0 | 75.0 | 86.4 | 0.90 |
| 75^th^ percentile | 100.0 | 66.7 | 84.6 | 77.8 | 100.0 | 87.2 | 0.87 |
| 90^th^ percentile | 85.7 | 83.3 | 84.6 | 85.7 | 83.3 | 91.0 | 0.88 |
| Mean | 71.4 | 100.0 | 84.6 | 100.0 | 75.0 | 86.7 | 0.90 |
| Skewness | 42.9 | 100.0 | 69.2 | 100.0 | 60.0 | 0.26 | 0.57 |
| Kurtosis | 57.1 | 83.3 | 69.2 | 80.0 | 62.5 | 1.53 | 0.67 |

*AUC*, area under the curve; *NPV*, negative predictive value; *PD*, proton density; *PPV*, positive predictive value
